# Provocative discography: Current status

**DOI:** 10.2349/biij.1.1.e2

**Published:** 2005-07-01

**Authors:** WCG Peh

**Affiliations:** Programme Office, Singapore Health Services, 7 Hospital Drive, #02-09, Singapore 169611, Republic of Singapore

**Keywords:** Discography, Low back pain, Intervention

## Abstract

Low back pain is a common clinical problem that may be due to a variety of causes, including disc disease. Provocative discography is an imaging-guided procedure in which a contrast agent is injected into the nucleus pulposus of the disc. Despite its controversial history, it remains the only imaging technique that provides both anatomical and functional information about a diseased disc. Disc morphology is usually assessed on either radiographs or computed tomography (CT), or both. Functional evaluation of the disc consists of pain provocation and careful assessment of the patient's response to pain. As provocative discography is an invasive procedure, it should not be used as a screening study in patients with back pain. It should instead be reserved for carefully- selected patients whose painful symptoms cannot be explained by findings on non-invasive imaging modalities such as magnetic resonance imaging or CT, and who are not responsive to conservative measures. Discography is helpful in selection of patients and disc levels to be operated upon. Careful application of indications and meticulous technique are however required if a successful outcome is to be expected.

## INTRODUCTION

Provocative discography is an imaging-guided procedure in which a contrast agent is injected into the nucleus pulposus of the intervertebral disc. It provides both anatomical and functional information about a disc suspected to be diseased. Following intradiscal contrast injection, disc morphology is usually assessed on radiographs or computed tomography (CT), or both. The functional evaluation consists of pain provocation and careful assessment of the patient's response to pain. The discography results influence the surgical decision-making process and selection of disc levels to be operated on.

Low back pain is a very common clinical problem. It may result from a variety of causes, including intervertebral disc disease. Currently, magnetic resonance (MR) imaging is widely regarded as the imaging modality of choice for investigating patients with suspected disc lesions. However, it is well known that many asymptomatic discs appear abnormal on MR imaging [1,2,3,4,5,6,7]. Discs that appear normal on MR imaging have also been shown to be abnormal on discography [8,9].

Ever since its first description in 1948, provocative discography has been regarded as a controversial procedure. To date, provocative discography remains the only imaging technique that directly relates the patient's pain response to the morphological appearance of the disc [10,11,12,13,14,15]. Despite an incomplete understanding of the pathophysiology of discogenic pain and the variable pain response of individual patients [16], many studies have supported provocative discography as a valuable diagnostic test in the investigation of discogenic pain [1,17,18,19]. However, being an invasive procedure, provocative discography should not be used as a screening study in patients with back pain but should instead be reserved for carefully selected patients.

## INDICATIONS

In general, provocative discography should be performed only if the patient has failed adequate attempts at conservative management of persistent severe back or neck pain and if non-invasive tests, such as MR imaging, do not provided sufficient information for a definitive diagnosis. To keep things in perspective, only a minority of patients presenting with low back pain require imaging. Pain due to facetogenic, neoplastic, inflammatory and traumatic causes should be excluded first, initially using radiographs and if required, supplementation by CT. The persistent back pain should be at least four months in duration and non-responding, before provocative discography is considered [20,21]. Discography should only be performed on a patient under consideration for operation to assist in identifying the appropriate level for surgery [22].

Specific indications for provocative discography are [11,12,14,15,19,22]:

1. Further evaluation of a radiologically-abnormal disc for the full extent of abnormality or correlation of the abnormality with the clinical symptoms.
2. Investigation of persistent, severe symptoms that do not correlate with equivocal or inconsistent MR imaging or CT findings.
3. Determination of symptomatic disc levels in cases where MR imaging or CT shows disc disease at multiple levels.
4. Assessment of disc prior to fusion to determine if a disc within proposed fusion segment is symptomatic, and whether the adjacent discs are normal.
5. Assessment of disc prior to percutaneously-directed therapies such as intradiscal electrothermal therapy [23,24,25,26].
6. Assessment of patients prior to minimally-invasive surgery in order to confirm that disc herniation is contained, or to investigate contrast distribution before chemonucleolysis.
7. Assessment of post-surgical failed back syndrome of patients in whom MR imaging is non-diagnostic, including differentiating recurrent disc herniation from a painful pseudoarthrosis or identifying a symptomatic disc within a posteriorly-fused segment.

## CONTRAINDICATIONS

Contraindications to provocative discography are [12,15,22]:

1. Patients with a known bleeding disorder and those on anticoagulation therapy.
2. Pregnancy.
3. Systemic infection or skin infection over the puncture site.
4. Severe allergy to injectate, especially the contrast agent.
5. Previously-operated disc.
6. Solid bone fusion that does not allow access to the disc.
7. Severe spinal cord compromise at disc level to be investigated.

## TECHNIQUE AND EQUIPMENT

Before the start of the procedure, the patient should be interviewed about the type, location and nature of the pain, and any history of prior surgery. Pain drawings may be helpful in identifying the specific discs that are associated with the patient's painful complaints [27]. The patient's medical and imaging records should be carefully reviewed, and the MR images compared with radiographs to evaluate for possible level ambiguity due to a transitional lumbosacral segment. MR imaging should be assessed for overall disc morphology and to identify a normal disc that can be used as a control.

In obtaining informed consent, the patient needs to understand the purpose of the pain provocation test and its risks. The patient should fast for six to eight hours prior to the procedure. Giving an intravenous dose of prophylactic antibiotics is recommended. In some centres, a mild sedative is administered prior to the procedure, while others do not recommend sedation as the patient's response to pain reproduction may be affected. The patient should ideally be monitored by nursing staff during the procedure. Strict asepsis is mandatory, with the radiologist being fully scrubbed up and gowned.

Provocative discography is best performed in an interventional suite within the diagnostic radiology department. Biplane fluoroscopy is preferred but if this is not available, then high-quality C-arm fluoroscopy is an acceptable alternative. In some centres, CT is used to guide needle placement. For patients who are allergic to iodinated contrast agents, MR discography using intradiscal gadolinium-chelate has recently been found to be a viable alternative [28,29,30,31,32].

There are variations in the size and type of needles used by different centres and practitioners. Some practitioners advocate the single needle approach using a styleted needle that ranges in size from 18- to 22-gauge [22]. Many practitioners adopt the double-needle approach for the following reasons: lower rate of discitis [33], use of the thinner 26-gauge inner needle to decrease the size of puncture hole in the annulus fibrosis, and having a pre-shaped curve at the distal end of the inner needle to facilitate entry into centre of the L5-S1 nucleus. In the double-needle technique, the inner needle that enters the nucleus pulposus does not come in contact with the skin, contributing to a reduction in the infection rate.

The discography set that I use consists of a 21-gauge 12.5 cm long stainless steel spinal needle with stylet and a 26-gauge 16.0 cm long stainless steel spinal needle with stylet for thoracic and lumbar discography. A 20-gauge 6.35 cm long stainless steel spinal needle with stylet and a 26-gauge 8.9cm long stainless steel spinal needle with stylet are used for cervical and thoracic discography. A curved needle set consisting of a 21-gauge 10.0 cm long stainless steel straight needle with stylet, and a 26-gauge 15.0 cm long nitinol curved needle is preferred for the L5/S1 disc.

## CERVICAL AND THORACIC DISCOGRAPHY

**Figure 1:**
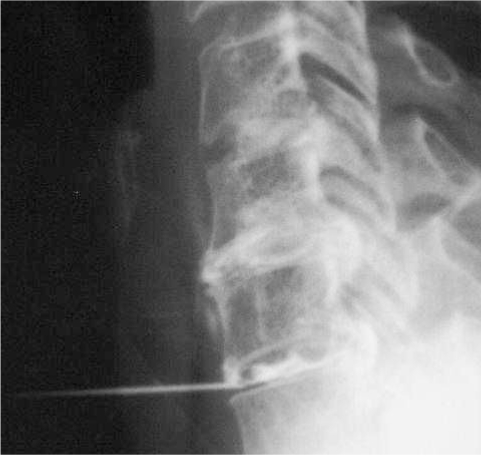
Cervical discography in a 49-year-old woman with neck pain. Lateral radiographic projection shows a normal C4/5 disc, and degenerate C5/6 and C6/7 discs with posterior protrusions. Note anterior approach used for needle placement. The needles for the upper 2 discs have been removed.

Cervical discography (Figure 1) remains a controversial procedure with some investigators recommending that this procedure should not be performed as the information obtained from cervical discography does not outweigh the increased risks of complications, reported to occur in up to 13% of cases [34]. These complications include discitis, epidural abscess, haematoma, myelopathy and quadriplegia [35]. Other practitioners have found cervical discography to be a safe and useful procedure in selected patients with chronic intractable neck pain with negative or indeterminate imaging findings, and are being considered for surgery [18,36,37].

There are very few indications for thoracic discography and it is rarely performed. Severe and disabling thoracic pain secondary to disc degeneration that requires discography has not been well studied [12,22]. This procedure has been used to evaluate symptomatic Scheuermann's disease [38]. Thoracic discs with prominent Schmorl's nodes may be intensely painful, even in asymptomatic subjects, and thoracic discography may demonstrate disc pathology that is not seen on MR imaging [39].

## LUMBAR DISCOGRAPHY

**Figure 2:**
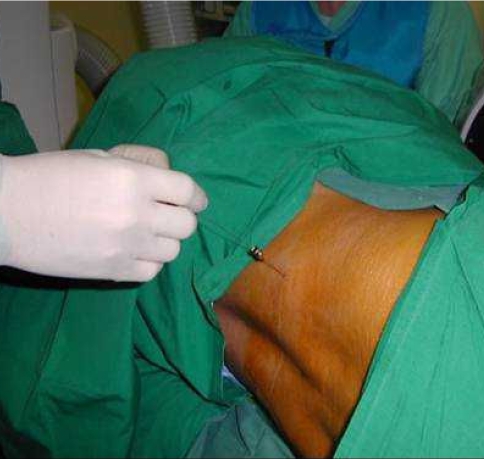
Patient positioning and skin puncture for lumbar discography. Photograph shows the patient lying in a left lateral decubitus position. The skin puncture point is approximately 8cm to the right of the midline. The shorter outer needle has been inserted with an obliquity of approximately 45 degrees to the sagittal plane. Its stylet is being removed in preparation for insertion of the longer inner needle.

The vast majority of discograms performed in clinical practice are for evaluating the lower three lumbar discs. For lumbar discography, the patient may be placed in a prone or left lateral decubitus position, depending on operator preference. Some advocate the prone position state in which the patient is more stable and immobile [12,22]. This author prefers the left lateral decubitus position. The patient flexes his or her knees to about 60° to 90°, with a pillow placed underneath his or her waist to keep the spine straight. The skin puncture point is approximately eight to 10 cm to the right of the midline. After the patient is cleaned and draped, and local anaesthesia is given, the outer discography needle is inserted (Figure 2).

**Figure 3:**
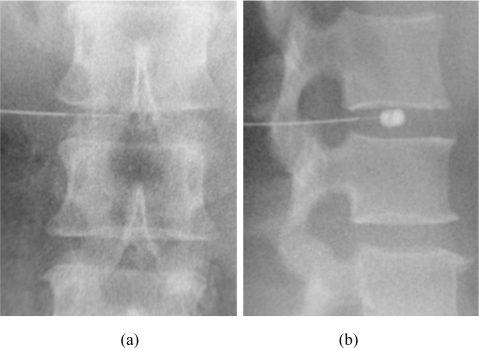
Needle placement for lumbar discography. (a) Anteroposterior and (b) lateral radiographic projections show the tip of the thicker, shorter outer needle at the posterolateral corner of the annulus fibrosis of the intervertebral disc. The tip of the thinner, longer inner needle is located in the centre of the intervertebral disc. Test injection of contrast agent confirms that the inner needle tip lies in the nucleus pulposus.

The posterolateral extradural approach is preferred as it avoids puncturing the thecal sac [12,15,22]. The outer needle is inserted with an obliquity of about 45° to 60° to the sagittal plane. For the L5-S1 disc, due to the overlying iliac crest, an additional caudal angulation of up to 40° is usually necessary. After repeated fluoroscopic imaging in the AP and lateral directions, the outer needle is positioned such that its tip is placed at the right posterolateral corner of the annulus fibrosis of the target disc. Imaging landmarks are: needle tip is located in line with the posterior cortex of the adjacent vertebral bodies on the lateral projection and in line with the ipsilateral pedicles of the adjacent vertebral bodies on the anteroposterior projection. Mild rubbery but firm resistance is felt when the needle tip comes into contact with the annulus fibrosis. The stylet of the outer needle is then removed, and the longer inner needle is inserted inside the outer needle. Under fluoroscopic guidance in the two orthogonal directions, the tip of the inner needle is directed to the centre of the nucleus pulposus (Figure 3).

When the position of the inner needle is satisfactory, its stylet is removed and the needle is attached to a 1 ml tuberculin syringe with 0.1 ml markings. A test injection of 0.1 ml of non-ionic contrast agent is then made to confirm the needle position (Figure 3b). The injected contrast agent should form a rounded or curvilinear blob near the centre of the disc space. In a normal disc, there is moderate resistance during contrast injection while in a degenerate disc, there is mild or no resistance to contrast injection. If there is marked resistance to contrast instillation at the beginning of the injection with the contrast agent staying immediately at the needle tip, then the needle tip may be located within the annulus fibrosis. If the position of the needle tip is suboptimal, adjustment of needle position and repeat fluoroscopic screening is required.

After the needles are removed, the patient's back or neck is cleaned, and small adhesive bandages are used to cover the puncture sites. Following completion of post-discography imaging, the patient should be observed for up to two hours in either a reclining or recumbent position. The patient's vital signs should be monitored. Upon discharge, most practitioners will give their patients a prescription of a non-narcotic painkiller, with an option of prescribing a short prophylactic course of oral broad-spectrum antibiotics [22].

## DISCOGRAPHY INTERPRETATION

The amount of contrast agent injected into the nucleus pulposus and resistance encountered during injection should be carefully recorded. The normal lumbar disc usually takes up to 1.5 ml of contrast agent. A degenerated lumbar disc will typically have a volume of more than 2 ml. Most practitioners would not inject more than 3 ml of contrast agent into a single lumbar disc. The volume of contrast agent injected should not exceed 0.5 ml per disc for cervical discography, while 0.5 ml to 1.0 ml is the usual volume for a normal disc in thoracic discography [12]. The injection is usually terminated when very firm resistance is felt or if severe pain is produced [22]. Discography interpretation may be supplemented by performing post-procedure imaging using CT (CT discography). The two major aspects to consider in the interpretation of discography are disc morphology and pain provocation.

**Figure 4:**
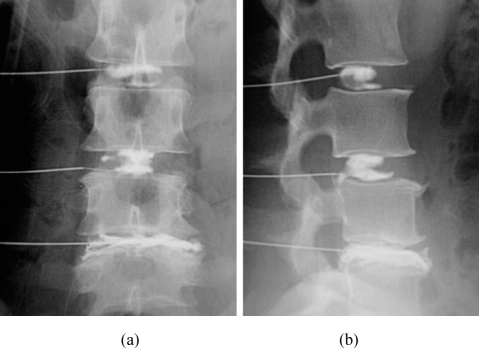
Discographic patterns in a 43-year-old woman who had low back pain with radiation to the left calf. (a) Anteroposterior and (b) lateral radiographic projections show a normal bilocular L2/3 disc. There is small posteroinferior tear of the L3/4 disc that was asymptomatic. The L4/5 disc is decreased in height, and had extensive annular disruption and posterior protrusion. The L4/5 disc was also symptomatic.

Disc morphology is usually determined on evaluation of anteroposterior and lateral radiographs obtained after intradiscal contrast injection (Figure 4). A normal disc maintains a normal height on both AP and lateral radiographs. Injected contrast agent remains in the nucleus pulposus, and may be unilocular (“cottonball” or rectangular) or bilocular (“hamburger bun”) in shape. Sometimes, a Schmorl's node is seen as focal protrusion of injected contrast agent into the adjacent vertebral end-plate [9].

In degenerated discs, discography shows a reduced disc height, and complex or multiple irregular fissures in the annulus fibrosis, with or without contrast leakage through annular tears. A bulging disc is often associated with degeneration, and is characterized by circumferential, diffuse and symmetrical annular bulging. Discography may show annular fissures with an intact peripheral annulus. Disc protrusion refers to focal, often asymmetrical, central or posterolateral protrusion of disc material within an intact posterior longitudinal ligament. On discography, a single annular fissure is often seen. The nuclear material may migrate superiorly or inferiorly (giving a “candle drip” appearance). A disc extrusion is a large disc protrusion that involves the posterior longitudinal ligament. On discography, an annular fissure with epidural space contrast extravasation is seen. A sequestrated disc is seen when extruded disc material is separated from the parent disc, with the detached disc being located in the extradural space.

**Figure 5:**
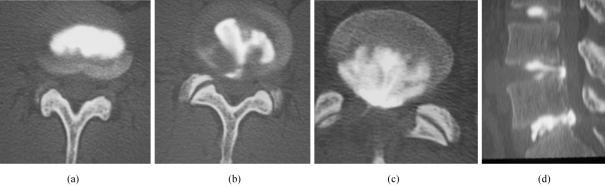
CT discographic patterns in a 36-year-old man who had low back pain with right buttock pain and right leg radiation. (a) Axial CT image shows a normal L3/4 disc (DDD grade 0). (b) Axial CT image shows a small L4/5 posterior annular tear (DDD grade 3). (c) Axial CT image shows extensive L5/S1 posterior annular disruption (DDD grade 4). (d) Sagittal recontructed CT image provides a good overview of a normal L3/4 disc, and posterior tears of protruding L4/5 and L5/S1 discs. Both lower discs were symptomatic.

CT discograms are CT images obtained following discography (Figure 5). It provides excellent anatomical details in the axial plane. The Dallas discogram description (DDD) is based on CT appearances and was originally classified into grades 0 to 3 [40], later modified to four grades [3]:

- **Grade 0**: Contrast agent is confined entirely within the normal nucleus pulposus (Figure 5a).
- **Grade 1**: Contrast agent extends radially along fissure involving the inner one-third of the annulus fibrosis.
- **Grade 2**: Contrast agent extends into the middle one-third of the annulus fibrosis.
- **Grade 3**: Contrast agent extends into the outer one-third of the annulus fibrosis, either focally or radially, to an extent not greater than 30° of the disc circumference (Figure 5b).
- **Grade 4**: Contrast agent extends into the outer one-third of the annulus fibrosis, dissecting radially to involve more than 30° of the disc circumference (Figure 5c).

Further modifications of the DDD are: **Grade 5** - representing a full-thickness tear, either focal or circumferential, with extra-annular contrast leakage [4]; **Grade 6** - representing disc sequestration; and **Grade 7** - representing a diffuse annular tear in disc degeneration [41]. Using a spiral or multislice scanner to perform the CT discogram produces good quality sagittal and coronal recontructed images that may be useful in providing additional information [42] (Figure 5d).

Discogenic pain is likely to be due to a combination of different mechanisms, all causing stimulation of nerve fibres located in the outer annulus fibrosis. The postulated mechanisms for discogenic pain provocation include stretching of fibres of the abnormal annulus fibrosis, extravasation of irritating chemical substances, pressure on nerves, vascularized granulation tissue in the annulus fibrosis, posterior joint hyperflexion during injection, and changes in the pattern of loading of the posterolateral annulus fibrosis or nucleus pulposus [43,44,45,46]. Where possible, injecting an adjacent normal disc as a control is recommended as it gives an indication of the patient's level of pain tolerance as well as the reliability of the patient's responses at other levels.

Pain provocation is the most useful and important aspect of discography. However, as the individual patient's response is subjective, it is important to avoid introducing bias during the procedure. Patients should instead be told before the start of the procedure and intermittently reminded to immediately inform the practitioner when they experience any new or increasing pain. Leading questions should be avoided. During injection, the location and character of the pain should be noted and recorded. It is useful to observe the patient's facial expression or body movement for signs of pain response.

The pain response can be classified into the following categories:

1. No or insignificant pain reproduction.
2. Pain different from the usual painful symptoms (discordant).
3. Pain similar to some of the usual painful symptoms (partially concordant).
4. Pain identical to the usual painful symptoms (concordant).

When taking the disc morphology and pain provocation aspects together, the categories of a discography study are:

1. Normal study.
2. Abnormal but asymptomatic disc(s)
3. Abnormal disc(s) with discordant symptoms.
4. Abnormal disc(s) with concordant (partially or fully) symptoms.

The finding of pain provocation during discography has been found to have a direct impact on the surgical outcome. Eighty nine percent of 137 patients with positive discograms had clinical benefit from subsequent operation [1]. There is a 75% surgical success rate in patients with both positive discograms and MR imaging at L5-S1 level, compared to only 50% success rate in patients with a combination of positive discograms and normal MR imaging [47].

## COMPLICATIONS

The complication rate of discography is low, and is accepted to be less than 1%. In a retrospective analysis of 10 discography studies in which prophylactic antibiotics were not given, an infection rate of 0.25% in 4891 patients and 0.094% in 12,770 discs was found, with the conclusion that the risk of post-discography discitis was minimal [48]. The most serious and frequently encountered complication is discitis. The incidence of infection can be decreased with the use of double needles, prophylactic antibiotics and styleted needles [11,33,49]. Many practitioners prophylactically administer broad-spectrum antibiotics as a precaution against possible discitis [12,15,22,49].

Nerve damage may also occur but usually causes only transient symptoms. Transthecal puncture route may result in post-procedural headache. Other possible complications are needle breakage, accidental intradural injection, intrathecal haemorrhage, meningitis, arachnoiditis, osteomyelitis, and epidural abscess. It has been shown that discography does not cause injury to the disc itself [50,51].

## CONCLUSION

Provocative discography remains the only diagnostic test that provides both anatomical and functional information about a suspected abnormal disc. It is a complementary test in patients whose painful symptoms are not explained by findings on non-invasive imaging modalities such as MR imaging or CT. Provocative discography is a helpful tool in the management of patients with low back pain, particularly for those who are not responsive to conservative measures. Careful patient selection and meticulous technique are paramount factors for a successful outcome.
